# Evaluation of Nile Tilapia (*Oreochromis niloticus*) Skin Peptides for Wound Healing: A Systematic and Meta-Analysis Review

**DOI:** 10.1021/acsbiomaterials.5c01219

**Published:** 2025-12-02

**Authors:** Rodrigo Tozetto, Julia B. de Macedo, Thais Leticia M. da Silva, Ana Carolina T. Ventura, Flávio Luís Beltrame, Priscileila C. Ferrari

**Affiliations:** Department of Pharmaceutical Sciences, State University of Ponta Grossa, General Carlos Cavalcanti Avenue, 4748, 84900-030 Parana, Brazil

**Keywords:** tilapia collagen, tilapia peptides, wound healing, regenerative medicine, preclinical studies, in vivo evaluation

## Abstract

Management of chronic wounds poses a significant challenge for medical teams worldwide, as it often requires prolonged hospitalization periods and frequently leaves sequelae, thereby becoming a public health problem. Furthermore, available medical treatments are usually ineffective for treating this type of injury; therefore, a survey for new treatments to achieve favorable outcomes is frequently sought. Among new treatment options for wound healing, the use of peptides extracted from the skin of Nile tilapia (*Oreochromis niloticus*) has shown favorable results. The objective of this work was to evaluate the evidence demonstrating the effectiveness of Nile tilapia skin peptides (NTSP) in modulating cellular and molecular mechanisms involved in the wound healing process in animal models. A systematic review and meta-analysis were performed using the PubMed, SciELO, Web of Science, and EMBASE databases. Articles from 2014 to 2024 were selected using a combination of keywords (tilapia skin) AND (wound healing) AND (peptides), along with synonyms, according to the MeSH criteria. A total of 378 studies were identified, of which 16 were deemed relevant based on the inclusion and exclusion criteria. According to the studies analyzed, NTSP delivery systems led to a decrease in the wound healing period, stimulated blood vessel formation, regulated and mediated anti- and pro-inflammatory cytokines, and controlled infection. Syrcle’s scale was used to assess the risk of bias, which was determined to be low. Additionally, the results from the meta-analysis demonstrate statistical significance in the findings from experiments utilizing NTSP. It is particularly evident in relation to wound retraction, wound closure, inflammatory score, and angiogenesis, indicating that the use of NTSP affects cellular and molecular mechanisms that stimulate the wound-healing process. However, significant heterogeneity was observed among the studies, which is a limitation of the analysis. Therefore, further clinical trials and standardized protocols are necessary to better elucidate the effects of NTSP.

## Graphical abstract

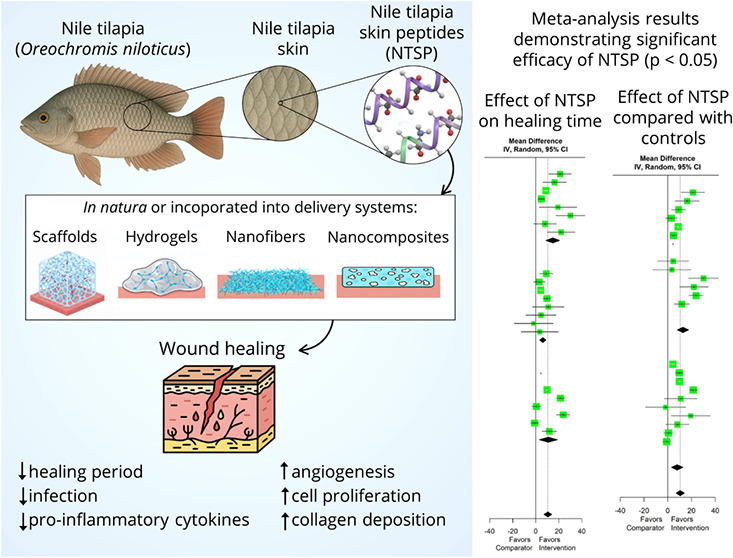

## 1 Introduction

The skin is the largest human organ, accounting for approximately 16% of body weight, and is capable of providing efficient physical defense against pathogens and allergens.^1^ The main events that cause loss and impairment of protective activity provided by the skin barrier are trauma, burns, and vascular complications of chronic diseases. These events lead to a decreased action of this natural protective shield, facilitating aggression from external agents, dehydration, secondary infections, delayed healing process, and sepsis.^2^

According to the World Health Organization (WHO), burns are responsible for more than 180,000 deaths annually worldwide, while those who survive may develop complex wounds with long periods of hospitalization and serious sequelae.^3^ Furthermore, it is estimated that 1 to 2% of the world’s population suffers from complex wounds resulting from venous diseases and arterial insufficiency of the lower limbs, resulting from complications of chronic diseases, such as type-2 diabetes.^4^

A complex wound is a term used to describe wounds that do not heal conventionally. These wounds challenge medical and nursing teams because they persist for a long time and do not respond to conventional treatments due to local or systemic factors, resulting in a significant socioeconomic impact. Treatment of complex wounds encompasses both clinical and surgical methods, utilizing topical ointments and dressings. Synthetic or biological materials are the most frequently employed treatments to aid tissue repair and control infection.^5^ Additionally, products such as gauze, lint, natural or synthetic bandages, and cotton wool are still used in some hospitals as primary or secondary wound dressings.^6^

The relative ineffectiveness of available topical products and the high costs of dressings made from synthetic or biosynthetic materials have led to increased research into biological materials derived from natural matrices, which offer lower costs, as an alternative for treating complex wounds.^7^

In this context, nature Nile tilapia (*Oreochromis niloticus)* skin and bioctives extracts of it like peptides have demonstrated acceleration in the healing process, control of secondary infections, and improvement in quality of the newly formed tissue, incorporating itself into the extracellular matrix (ECM) of the recipient, and temporarily providing coverage of epidermal defect caused by trauma, until damaged tissue is repaired.^8^

Nile tilapia is a globally farmed freshwater fish valued for its rapid growth, adaptability, and nutritional quality. Its production contributes significantly to income generation, commercial development, and food security by providing high-quality protein and essential nutrients. In 2020, global tilapia production was estimated at nearly 7 million tons. Despite its economic relevance, the skin is generally discarded as a byproduct of the production chain. To add value to this waste material, researchers have explored its biomedical potential, identifying its high biocompatibility and morphological similarity to human skin, including a high content of type I collagen and several bioactive compounds.^9,10^

The bioactive composition of Nile tilapia skin extends beyond collagen, which provides structural support, promotes tissue regeneration, and contributes to ECM formation. It contains hydrophobic amino acids such as glycine, proline, alanine, valine, and hydroxyproline, which serve as precursors to bioactive peptides with antimicrobial, antioxidant, and immunomodulatory properties. In addition, glycoproteins and proteoglycans contribute to hydration, ECM maintenance, and cell signaling; essential minerals support cell proliferation and tissue repair; and small amounts of lipids and natural antioxidants exert anti-inflammatory and protective effects.^10,11^

Specifying, studies indicate that Nile tilapia skin peptides (NTSP) accelerate the healing process through distinct molecular mechanisms, such as inhibition of the production of Tumor Necrosis Factor-α (*TNF-α*), inhibition of pro-inflammatory cytokines such as Interleukin-1 (*IL-1*), Interleukin-6 (*IL-6*), Interleukin-8 (*IL-8*) and through other pathways involved in healing processes such as regulation of antimicrobial peptide (*BD14*), Fibroblast Growth Factor-β (*FGF-β*) and Vascular Endothelial Growth Factor (*VEGF*).^12,13^

Furthermore, peptides extracted from NTSP have demonstrated antimicrobial activity by directly affecting the metabolism of pathogenic microorganisms and exerting regulatory effects on the innate immune system. They also positively modulate the healing process and exhibit antioxidant activity.^14^ Therefore, developing a natural product that uses NTSP as an alternative for treating skin wounds is justified, as there are treatment options for complex wound management. Given that the results of several studies indicate its healing, antimicrobial, antioxidant, and immunomodulatory properties.^15^

To motivate investment and development of dressings using NTSP for medical use in conventional wound healing and as an alternative treatment for complex wounds, this work comprises a systematic literature review of important databases. The objective is to evaluate the therapeutic efficacy of this natural matrix at the cellular and molecular levels in animal models, elucidate the delivery systems that transport these active compounds, compare them to existing medical treatments, and analyze their benefits and limitations in both experimental settings, including *in vitro* and *in vivo* studies.

## 2 Methods

### 2.1 Standardized Criteria, Protocol, and Registration

This systematic review and meta-analysis adhered to the criteria for preparing systematic reviews and meta-analyses outlined in the Cochrane Handbook for Systematic Reviews of Interventions (version 5.1.0)^16^ and the PRISMA (Preferred Reporting Items for Systematic Reviews and Meta-Analyses) guidelines. The study protocol was registered at the International Prospective Register of Systematic Reviews (PROSPERO; CRD42024589083; date of registration: 12 November 2024).

### 2.2 Eligibility Criteria

The primary question guiding this study was: “Compared to conventional wound treatments, are Nile tilapia skin peptides effective in accelerating wound healing, with potential applications in medical treatments?”

The PICO strategy guided the analysis:

1. Population: Animal models for treatment of skin lesions.
2. Intervention: Treatment of skin lesions with Nile tilapia skin peptides or Nile tilapia skin collagen.
3. Comparison: Treatment using control groups (negative or conventional methods or available medical products).
4. Outcome: Treatment effectiveness concerning the stages of wound healing, such as inflammation, proliferation, and/or remodeling.

### 2.3 Inclusion/Exclusion Criteria

#### 2.3.1 Inclusion Criteria

It selected original articles published in Portuguese, English, and Spanish between 2014 and 2024 and applied the following inclusion criteria: articles published in full; studies that included *in vivo* and *in vitro* trials; that evaluated the therapeutic response to the use of Nile tilapia skin as well as collagen peptides in its composition; that performed topical treatment of skin wounds induced by surgical excision, burns or other types of injuries; that evaluated the efficacy of treatment with peptides concerning conventional treatments and other comparators; that used an animal model in the research.

#### 2.3.2 Exclusion Criteria

Thesis, literature reviews, and other nonoriginal articles were excluded from this study; those that do not use *in vivo* or *in vitro* models; clinical trials with human participants; studies that do not compare the results of treatments using collagen peptides with conventional treatments or other comparators; studies that use other animal sources of collagen in the treatment such as swine, bovine; studies that use collagen peptides from other fish species.

Clinical trials in humans were intentionally excluded because most research on NTSP remains at the preclinical stage, and the few available clinical reports are recent, heterogeneous, and not yet supported by standardized industrial processes that guarantee reproducible safety and efficacy comparable to approved dressings. Thus, we aimed to synthesize mechanistic and translational preclinical evidence.

### 2.4 Study Search Strategy

Data were collected using PubMed, SciELO, Web of Science, and EMBASE platforms. Words “Tilapia Skin”, “Wound Healing” and “Peptides” were used as Health Sciences Descriptors (DeCS), and the following search strategies were used: [(tilapia skin) OR (fish skin)] AND [(wound healing) OR (complex wound) OR (wound dressing) OR (skin regeneration) OR (injuries) OR (burn healing) OR (tilapia topical treatment)] AND [(peptides) OR (collagen peptides) OR (tilapia piscidin) OR (collagen powder) OR (collagen fibers) OR (collagen peptides mixture) OR (hydrolyzed gelatin peptide) OR (hydrolyzed Collagen)].

### 2.5 Data Collection Process

The selection of articles and data collection were performed by two previously calibrated reviewers (RT and JBM); disagreements were resolved by a third reviewer (TLMS). Additionally, a researcher with specialized training in wound healing treatment (PCF) provided clinical and research support for data collection, discussed topics addressed, and offered expertise in the area.

### 2.6 Extracted Data Items

As described in the flowchart (Figure 1), in the initial search of the identification phase, 378 articles were found in all databases, and duplicate articles (*n* = 114) were excluded. The articles selected in this first stage (*n* = 264) underwent title screening, and (*n* = 168) articles that did not meet the determined criteria were eliminated. Thus, the (*n* = 96) included articles had their abstracts analyzed, excluding (*n* = 51) works.

**Figure 1:**
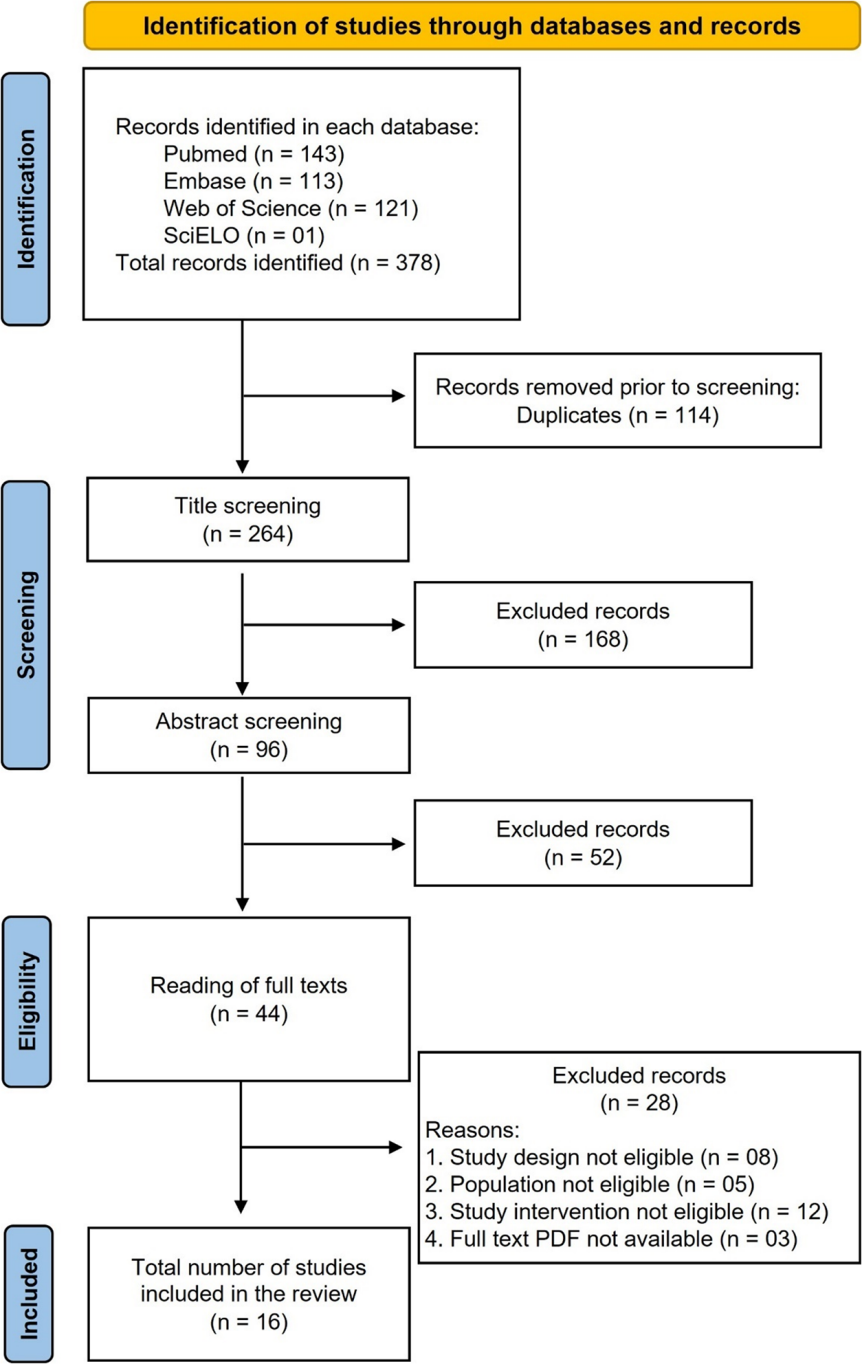
PRISMA flowchart represents the entire process of searching and selecting database articles.

Thus, the (*n* = 44) articles that met the criteria underwent a meticulous reading of their full texts, and after deliberation by four research group members, the articles were selected to compose the present study. The final sample of this review consisted of 16 articles, all of which were randomized clinical trials that strictly met the inclusion and exclusion criteria.

In the next phase of the review, the studies were analyzed, and the data were grouped in an organized and synthesized manner through the construction of a synoptic table containing the following data: authors, title, journal year, type of study, animal model, intervention, comparators, outcome and main results obtained, in order to extract the data in an organized manner for the discussion and meta-analysis.

The interest variables analyzed *in vivo* assays were wound retraction, histological analysis, inflammatory infiltrate, blood vessels, and collagen fibers. Additionally, molecular markers such as *VEGF*, Transforming *TGF-β1*, and inflammatory cytokines, including *IL-6*, were examined. The *in vitro* assays have examined various variables, including cell proliferation and migration of keratinocytes (HaCaTs), fibroblasts (HDFs), murine cell derivatives (MC3T3-E1), murine fibroblasts (L929), lymphocyte proliferation, hemolysis, antimicrobial activity, toxicity, and biocompatibility. It also analyzed the development of delivery systems to carry NTSP and discussed the main results of delivery systems developed for wound treatment.

### 2.7 Evaluation of the Study Quality and Risk of Bias

According to Brazilian National Health Council Resolution 466/12, submitting this work to the Research Ethics Committee was not necessary. This project risks plagiarism and interpretation bias; however, the authors are committed to avoiding plagiarism and discussing all articles jointly, thereby minimizing the risk. This review was assessed using the SYRCLE scale regarding the risk of bias.

The SYRCLE scale (SRs) was used to assess bias and level of evidence; since animal intervention studies differ from randomized clinical trials (RCTs) in many aspects, the SRs methodology for clinical trials must be adapted and optimized for animal intervention studies. Thus, under the supervision of Cochrane, an international nonprofit organization dedicated to developing, maintaining, disseminating, and establishing criteria for conducting systematic reviews, a Risk of Bias (RDV) tool was developed to ensure consistency and avoid discrepancies in assessing the methodological quality of RCTs. The resulting RDV tool for animal studies comprises 10 questions related to selection bias, performance bias, detection bias, attrition bias, reporting bias, and other biases that enhance the reliability and applicability of the extracted data.^17^

### 2.8 Outcomes

The primary outcome was based on the degree of wound healing in model animals treated with Nile tilapia skin peptides or Nile tilapia skin collagen compared to negative control or conventional treatments. The measures were evaluated using healing parameters to assess the efficacy of the studies. The secondary outcome was based on in vitro studies of cell migration, cytotoxicity, and antimicrobial activity after treatment with Nile tilapia skin peptides or collagen, compared to conventional formulations.

### 2.9 Statistical Analyses and Meta-Analysis

Meta-analyses were performed to compare the results of intervention groups (any intervention using tilapia) with their comparators (those with and without an active ingredient). Not all studies were included in this analysis, and parameters for which results were obtained from at least two studies were wound closure (%), wound retraction (%), scar retraction area (mm^2^), inflammatory cells, *IL-6*, inflammation score, lymphocytes (%), *TGF-β1*, and blood vessels (%). The inverse variance method and the calculation of the mean difference (MD) were used to estimate how the intervention changes the average result compared to the control. Heterogeneity between results was tested using the *I* ^2^ statistic, and when significant (*p* < 0.05), random-effect models were employed. When it was not significant, fixed effects models were used.

Publication bias was assessed using the Egger test, with significance defined as *p* < 0.05. Given the differences between the studies, subgroup analyses were performed considering groupings according to animal species (mouse, rat, rabbit), evaluation times (up to 7 days, 8 to 14 days, 15 to 21 days, and over 21 days postintervention), application form (liquid, gel, powder) and comparator group (with and without active ingredient). The results were presented with the summary measure obtained, its 95% confidence intervals, and Forest plot-type graphs. For this analysis, the meta package^18^ was used in the R 4.1.0 environment.^19^

## 3 Results

### 3.1 Peptides Extraction

Tilapia skin has been explored as a wound dressing since its first use in a human clinical trial in Brazil in 2017,^20^ and can be used in its natural state,^20,21^ decellularized,^22^ or treated to extract the NTSP (Table 1).

**Table 1:** Summary of Peptide Extraction Methods and Their Characteristics^a^

Tilapia peptide	peptides extraction	preparation and purification	characterization analysis	peptides characteristics	reference
Raw hydrolyzed extract	Alkali treatment with NaOH (10% (w/v), pH 8.25) and enzymatic reaction with *Bacillus licheniformis* alcalase (0.85 g).	Liofilization and mixing with potassium sorbate (0.2%).	Composition and molecular weight: Mass spectroscopy.	Twenty peptide sequences were identified, including di- and tripeptides.	^23^
Raw hydrolyzed extract	Tilapia homogenization with distilled water (1:3, w/v), centrifugation, and lyophilization. Resuspension of lyophilized extract in buffer solution (1:1, v/v), centrifugation, enzymatic reaction of the supernatant with trypsin enzyme (1:0.2, v/v), and incubation at 38 °C at different times for digestion.		Composition and molecular weight: Mass spectroscopy.	Sixteen proteins were identified, including type I collagen peptides (α 1, 2, and 3) and low-molecular-weight peptides (<5 kDa); 9 essential (11.76%) and 30 nonessential amino acids (88.24%), predominantly hydrophilic (>56%), glycine (33.6%), proline (16%), and alanine (16%).	^24^
			Amino acid content: PEAKS software and e ProtParam-ExPASy tool.		
Collagen peptides	Enzymatic reaction with neutral protease and papain, heated to 50 °C for 5 h, then heated to 100 °C for inactivation, followed by centrifugation.	Filtration (50 nm ceramic membrane), concentration under reduced pressure, and spray-drying of the supernatant.	Composition and molecular weight: HPLC.	Low-molecular-weight polypeptides (99.14% < 5 kDa); 7 essential (16.18%) and 10 nonessential amino acids (79.56%), predominantly hydrophilic (>58%), glycine (20.92%), proline (11.32%), and hydroxyproline (10.28%); random coil conformation.	^25^
			Amino acid content: Amino Acid Analyzer.		
			Chemical structure: FTIR.		
Chitosan-Marine Peptides	Heating tilapia skin in distilled water at 100 °C for 10 min, homogenization, dilution in water at 1:3 (w/v), transferred to a hemolytic reactor, enzymatic reaction with neutral protease and papain for 4 h, heated to 100 °C for 10 min, centrifugation.	Filtration (50 nm ceramic membrane), evaporation on a rotary evaporator, and spray-drying of the supernatant.	Composition and molecular weight: HPLC and electrospray ionization-mass spectrometry.	98.77% had a molecular weight of 1000 Da, and 1.23% among 1000–3000 Da; 351 amino acid sequences were identified, 92.3% had a length of 8 amino acids; 8 essential and 9 nonessential amino acids, glycine (20.4%), proline (10.8%), and hydroxyproline (11.2%).	^26^
			Amino acid content: Amino Acid Analyzer.		
Type I collagen powder	Removal of fat with deionized water, isopropanol (10%, v/v), NaOH (0.1 mol/L) solution + *n*-hexane, and Triton solution (0.3%, v/v), followed by soaking at 8 °C for 24 h. Enzymatic reaction with enzyme solution (3.5%, v/v) (not specified), at 1:21 (w/v), for 14.5 h.	Liofilization and liquid nitrogen freeze-grinding.	Morphology: optical and SEM.	Morphology: lamellar structures with filamentous structures interspersed, irregular sheet-like folding structure, large pores (50–150 μm), wide distribution, and microfiber structure; composed of predominantly and complete α1 and α2 chains, >100 kDa; triple helix conformation. Denaturation temperature: 57.5 °C.	^27^
			Composition and molecular weight: SDS-PAGE.		
			Chemical structure: ultraviolet spectroscopy (200–400 nm) and FTIR.		
			Secondary and internal structure: circular dichroism spectroscopy and DRX.		
			Thermal stability: DSC.		
Type I collagen sponge	Alkali treatment with NaOH (0.1 M), followed by acid dissolution with acetic acid (0.5–1 M) and enzymatic reaction with pepsin (0.1–0.5%, v/v). Collagen was precipitated with ammonium sulfate (0.4 M).	Dissolution of the precipitate in acetic acid, dialysis, and lyophilization.	Composition and molecular weight: SDS-PAGE.	Composed of α-chains α1 (132 kDa) and α2 (119 kDa); cross-linked chains β (278 kDa) and γ (300 kDa); 19 amino acids, glycine (31.9%), proline (11.3%), and hydroxyproline (7.7%). Denaturation temperature: 33.99 °C.	^28^
			Amino acid content: Amino Acid Analyzer.		
			Thermal stability: DSC.		
Type I collagen sponge	Method of acidase combination (technique not reported).	Liofilization.	Morphology: SEM.	Morphology: sponge with honeycomb-like porous, with pore sizes of 20–120 μm, good elasticity, and a water absorption rate of ∼1900% in the dry state.	^29^
Type I collagen peptides	Removal of noncollagenous proteins with 20 volumes NaOH (0.1 N) at pH 12, stirring for 4 h, followed by acid dissolution with 1:70 (w/v) acetic acid (0.5 M) at 4–6 °C for 24 h, centrifugation, and salting out the supernatant by adding NaCl (0.9 M).	Dissolution of the precipitate in acetic acid, dialysis, and lyophilization.	Chemical structure: FTIR.	Triple helix conformation.	^30^
Type I acid-soluble collagen	Removal of noncollagenous proteins with NaOH (0.1 M) at 1:10 (w/v). Removal of fat with butanol (10% v/v) at 1:10 (w/v) for 24 h. Acid dissolution with acetic acid (0.5 M) at 1:50 (w/v) for 96 h, salting out the supernatant by adding NaCl (2 M).	Dissolution of the precipitate in acetic acid, dialysis, and lyophilization.	Composition and molecular weight: SDS-PAGE.	Composed of α-chains α1 (130 kDa) and α2 (120 kDa); cross-linked chains β (280 kDa) and γ (300 kDa); predominant amino acids were glycine (27.85%), proline and hydroxyproline (12.87%), rich in glutamic acid (10.80%) and alanine (10.37%); triple helix conformation. Denaturation temperature: 32 °C.	^31^
			Amino acid content: Amino Acid Analyzer.		
			Chemical structure: FTIR.		
			Internal structure: DRX		
			Thermal stability: DSC.		
Piscidin 3 (TP3)	Peptides were synthesized by GL Biochem (Shanghai, China) by Fmoc Solid-Phase Peptide Synthesis. Crude peptides were extracted (the specific extraction method was not reported).	Liofilization and HPLC.	Purity: HPLC.	TP3 amino acid sequence: FIHHIIGGLFSVGKHIHSLIHGH	^14^
			Composition and molecular weight: Mass spectroscopy.		
Piscidin 2 (TP2–5 and TP2–6)	Synthesized by GL Biochem (Shanghai, China) (method not reported).			Amino acid sequence:	^4^
				TP2: GECIWDAIFHGAKHFLHRLVNP	
				TP2–5: KKCIAKAILKKAKKLLKKLVNP	
				TP2–6: KKCIAKAILKKAKKLLKDLVNP	

a HPLC: High-performance liquid chromatography; FTIR: Fourier transform infrared spectroscopy; SEM: Scanning electron microscopy; SDS-PAGE: sodium dodecyl sulfate-polyacrylamide gel electrophoresis; DRX: X-ray diffraction; DSC: Differential scanning calorimetry.

Collagen, the main component of the ECM, represents the most prominent class of NTSP, exhibiting good biocompatibility, low immunogenicity, biosafety, and mechanical properties.^29^ It has a protein structure characterized by a Gly-X-Y repeating sequence, with glycine, proline, and hydroxyproline or alanine being the most abundant amino acids.^23,29^

Collagen peptides can be extracted from tilapia skin through enzymatic reactions, which enhance their therapeutic properties by reducing the molecular weight of the matrix components in relation to the existing collagen molecule.^23^ Tozetto et al. utilized *Bacillus licheniformis* alcalase to produce a raw hydrolyzed extract of tilapia skin, obtaining di- and tripeptides. This demonstrates that enzymatic hydrolysis efficiently generates smaller and more biologically active peptides.^23^ This was corroborated by Cardoso et al., who extracted the collagen using the enzyme trypsin and obtained low-molecular-weight NSTP (<5 kDa), which was interestingly the most hydrophilic (>56%).^24^

Similarly, Hu et al. utilized neutral protease and papain to extract collagen from tilapia skin, yielding low-molecular-weight polypeptides with over 58% of the total amino acids being hydrophilic residues, a property that could potentially improve histocompatibility. The infrared spectrum showed that the main molecular conformations within the collagen peptides were predominantly random coil.^25^ Ouyang et al. also employed neutral protease and papain to extract collagen, obtaining 98.77% low-molecular-weight NTSP with good aqueous solubility. A total of 351 sequences were identified, and 92.3% of them had a length of 8 amino acids, which favors skin absorption.^26^

Furthermore, Yang et al. produced collagen hemostatic powder from tilapia skin using enzymatic extraction, followed by lyophilization and liquid nitrogen freeze-grinding. These techniques preserved the collagen’s original triple-helix structure and maintained the integrity of the peptide chains, as the low temperature prevented protein denaturation.^27^

In addition to enzymatic reactions, collagen can be extracted and isolated from tilapia skin using acid/alkali treatments or through a combination of both methods. For example, Zhou et al. combined two methods to extract collagen from tilapia skin: acid dissolution treatment, which preserves the triple helix structure of collagen to the greatest extent, and pepsin digestion, which reduces antigenicity by removing the N-terminal and C-terminal regions of the peptides. This extraction method yielded type I collagen structure, mainly composed of high-molecular-weight polypeptides. After extraction, the product was lyophilized to obtain collagen sponges.^28^

Similarly, Wang et al. extracted tilapia collagen using a combination of the acidase method. The collagen concentrate was placed into a mold and freeze-dried to obtain collagen sponges.^29^ However, their study focused solely on the physical characterization of the sponges, without providing details on their chemical composition or structure.

Elbialy et al. employed acid and pepsin treatments to extract collagen from tilapia skin. Fourier transform infrared spectroscopy (FTIR) analysis confirmed the presence of amide A, B, I, II, and III bands, demonstrating that the extracted collagen maintained its triple-helical structure and characteristic amino acid profile.^30^ Mukta et al. used the same treatment to extract acid-soluble collagen from tilapia skin and obtained collagen with comparable characteristics, supporting the reliability of acid and pepsin solubilization methods for preserving the native structural and biochemical properties of tilapia-derived collagen.^31^

Beyond collagen, tilapia produce marine antimicrobial peptides (MAPs) as part of their immune system, such as piscidin, defensin, hepcidin, cathelicidin, and histone, which have antimicrobial effects and are essential for keeping the wound area free from infection.^24^ Piscidins are a family of cationic antimicrobial peptides expressed by fish mast cells, consisting of structurally related mature amphipathic α-helical structure peptides of 21 to 44 residues.^14^

In Nile tilapia, five piscidin-like peptides were identified and named tilapia piscidin (TP). Among them, tilapia piscidin 3 (TP3) is a 23-amino acid pore-forming peptide with an α-helix structure, which starts with phenylalanine and ends with histidine.^14^ However, the relatively high cytotoxicity and hemolytic activities of TP3 and TP4 peptides have limited their clinical application.^4^ To overcome this limitation, the Liu et al. research group developed two peptides derived from the TP2 sequence, called TP2–5 and TP2–6, which retained the antimicrobial properties while reducing adverse effects.^4^ These types of NTSP are often chemically synthesized, as performed in these studies by GL Biochem (Shanghai, China).^4,14^

Protocols for the extraction and characterization of NTSP vary considerably across the included studies. Furthermore, the lack of standardized protocols for peptide preparation and quality control introduces a significant source of heterogeneity in the reported outcomes. Differences in hydrolysis conditions, such as enzyme type, temperature, pH, and reaction time, can directly influence the resulting peptide profiles, including chain length, amino acid composition, and molecular weight distribution.^32–34^

### 3.2 Qualitative Results

#### 3.2.1 In Vivo Studies

NTSP has demonstrated, in several trials, the ability to accelerate tissue regeneration and wound healing through a series of mechanisms, including the mobilization of fibroblasts, modulation of cytokines and growth factors, as well as stimulation of angiogenesis and collagen fiber production. From selected articles, 15 (*n* = 15) evaluated wound closure, four (*n* = 4) neovascularization, four (*n* = 4) inflammatory cell infiltration, two (*n* = 2) presence of fibroblasts in scar tissue, five (*n* = 5) collagen fibers, and 11 (*n* = 11) performed immunohistochemical analysis (Table 2).

**Table 2:** Summary of results of included studies (*n* = 16)

animal model	intervention	comparators	main results	reference
Male mice (C57) (n = 30), 4–5 months old. Surgical flap excision (1.2 cm) in diameter	Topical application of peptide T19 in concentrations of 18.75 and 37.5 μg/mL extracted from NTSP for 9 days	Distilled water	Greater scar retraction and neoformation of blood vessels were observed in the NTSP group, along with increased recruitment of anti-inflammatory mediators such as *IL-2*, *IL-4*, and *CD-31*, as well as regulation of pro-inflammatory mediators, including *CD-163*, *COX-2*, *K*_*i*_ *-67*, and *PCNA*.	^24^
*Rattus norvegicus* (*n* = 16) males, 2–3 months old. Surgical flap excision (1.5 × 1.5 cm^2^)	Topical application of collagen gel extracted from NTSP for 15 days	Dressing without the active ingredient	Greater scar retraction on all days tested (days 3, 6, 9, 12, and 15), greater recruitment and activation of macrophages, fibroblasts, and angiogenesis through the positive regulation of the expression of the genes *TGF-β1*, *FGF-β*, and *α-SMA*, and greater expression of *TGF-β1* and *VEGF* for the NTSP group.	^30^
Male mice (C57) (*n* = 32), 3–4 months old. Scalding Burn (2 × 3 cm^2^)	Topical application of fresh NTSP over the wound, 28 days	Hydrocolloid adhesive bandage	*VEGF* and *FEGF* values corroborate these findings, with these markers significantly elevated on day 16 in the group treated with NTSP.	^20^
New Zealand rabbits (*n* = 48), males and females, 3–4 months old. Scald wounds (4 cm^2^)	Topical application of lyophilized NTSP, 28 days	Topical petroleum jelly	Wound healing in rabbits was faster and more effective for treatment with NTSP. Additionally, the *in vitro* scratch assay (Scratch Assay) for treatment with NTSP showed the best cell migration results for the NTSP group..	^25^
Mice (Balb C) (*n* = 35), 3–4 months old. Surgical flap excision (1.0 cm diameter)	Topical application of NTSP TP3 (2 mg/mL), 28 days	Methicillin and vancomycin	Reduction in healing time, low toxicity, and lower mortality in rats infected with multiresistant bacteria. Furthermore, the most significant reduction in bacterial load in the wound was observed with NTSP treatment.	^14^
Spregue Rats Dawley (*n* = 36), male and female Bama mini pigs (*n* = 10), 2–3 months old. Excision 3 flaps 1.8 cm diameter (rats)/Excision Flap 5 × 5 cm^2^ (pigs).	Application of acellular collagen dermal matrix with NTSP, 21 days	Acellular dermal matrix with porcine peptides and petroleum jelly	Improved wound healing, increased migration of keratinocytes and fibroblasts, stimulation of collagen synthesis and *KGF* expression in fibroblasts, promotion of endothelial cell migration and angiogenesis in the NTSP group.	^22^
Male mice (Balb C) (*n* = 32), 4 months old. Punch excision (0.5 cm) in diameter	Topical application of NTSP TP2–5 and TP2–6, 10 days	EGF and PBS Vehicle	Acceleration of wound closure in an animal model with promotion of proliferation and migration of keratinocytes and fibroblasts, stimulation of collagen synthesis, and expression of *KGF* in fibroblasts. Additionally, it leads to greater migration of endothelial cells and enhanced angiogenesis in the group treated with NTSP.	^4^
Male *Rattus norvegicus* (*n* = 12), 2–3 months old. Punch excision (1 cm) in diameter	Scaffold application of electrospun NTSP for 14 days.	PLA polymers and negative control	Greater wound closure for the group treated with tilapia peptides on days 3 and 7; on day 10, there was already 100% wound closure, while the comparator group on day 14 still had 90% scar retraction in the NTSP group.	^31^
New Zealand rabbits (*n* = 27), males and females, 3–4 months old. Scald wounds (3 cm^2^)	Topical application of lyophilized NTSP + chitosan, 21 days	Ointment for burns and saline solution	Greater wound closure, antibacterial activity, migration of L929 cells, expression of vascular endothelial growth factor (*VEGF*), and fibroblast growth factor 2 (*FGF2*) for the group that received NTSP. Furthermore, this same group presented the lowest cytotoxic effects on L929 cells.	^26^
New Zealand rabbits (*n* = 20), males and females, 3–4 months old. Scald wounds 3 cm^2^	Application of NTSP/Chitosan/Hydroxyapatite hydrogel, 21 days	Mebo burn cream	Significant antibacterial activity, cytocompatibility, acceleration of burn healing, and total protein synthesis in granulation tissue. Reduction of the local inflammatory response of the wound, increased angiogenesis, and epithelialization. Promoted the production of collagen fibers and increased expression of *STAT3* and *VEGF* for the group treated with NTSP.	^35^
Male *Rattus norvegicus* (*n* = 24), 2–3 months old. Surgical flap excision (2 × 3 cm^2^)	NTSP graft (*in natura*), 7 days	Amniotic membrane and negative control	Greater wound retraction, greater re-epithelialization tissue thickness, less fibrosis formation, and better regulation of the healing process for the NTSP group.	^21^
Male *Rattus norvegicus* (*n* = 70), 3 months old. Surgical flap excision (2 × 2 cm^2^).	Topical application of Natrosol gel with NTSP in 3 concentrations, 21 days	Natrosol gel with bovine collagen and without the active ingredient	Greater wound closure effect, inflammatory response modulation, angiogenesis stimulation, fibroblast migration, and collagen fiber synthesis for the NTSP group.	^23^
Spregue Rats Dawley (*n* = 90), males, 2–3 months old. Surgical flap excision (1.5 × 1.5 cm^2^)	Topical application of NTSP sponge, 14 days	Bovine collagen sponge and antiseptic	Enhanced wound closure and vascular growth through *VEGF* and *FGF* expression, cell migration, and proliferation. Promoted collagen deposition and maturation in the wound bed through modulation of *FPKN I* and *III* in the group treated with NTSP sponge.	^29^
Spregue Rats Dawley (*n* = 54), males, 3–4 months old. Surgical flap excision (1.5 × 1.5 cm^2^)	Topical application with a dressing containing lyophilized collagen from NTSP, 21 days	Topical application of lyophilized bovine collagen	Greater wound closure of the wound area on days 7, 14, and 21 of the experiment, shorter time of hepatic hemorrhage, and lower cytotoxicity for fibroblasts in the group that received treatment with lyophilized collagen from NTSP.	^27^
Spregue Rats Dawley (*n* = 24), males and females, 3–4 months old. Excision of 3 surgical flaps (1.8 cm) in diameter	Application of NTSP nanofibers, 14 days	Application of alginate (Tegaderm)	Increased viability, migration, and differentiation of HaCaTs with increased expression of *MMP-9*, *TGF-ÿ1*, involucrin, filaggrin, and *TGase1*. In addition, increased expression of *Col-I* in *HDFs* and *TGF-ÿ1*. There is still a significant increase in re-epithelialization and dermal reconstruction in wound healing for the group treated with NTSP nanofibers.	^36^
Spregue Rats Dawley (*n* = 24), males, 3–4 months old. Flap excision (3 flaps) 1.8 cm diameter	Application of NTSP sponge, 14 days	Application of alginate (Tegaderm)	Acceleration of wound closure without induction of an immune response. In addition to greater adhesion, proliferation, and differentiation of HaCaTs with increased expression of involucrin, filaggrin, and *TGase1*. Furthermore, greater stimulation of re-epithelialization was observed in wounds of the group treated with NTSP.	^28^

##### 3.2.1.1 Wound Closure

Expressive wound retractions were observed by Cardoso et al. (2024) in T1 (18.75 μg/mL) and T2 (37.5 μg/mL) groups that used NTSP against a control group after 9 days of treatment (64.4 ± 10.23% and 59.5 ± 11.65% against 43.2 ± 14.27%).^24^ Additionally, Elbialy et al. (2020) observed a greater wound retraction in the NTSP gel group compared to the control (76.9 ± 12.45% and 59.3 ± 15.6%).^30^ Furthermore, Hu et al. (2017) obtained relevant results using NTSP compared to commercial ointment for burns (78.6 ± 11.1% and 70.5 ± 23.5%) in 14 days.^25^ Garrity et al. did not observe a statistical difference between the NTSP and hydrocolloid gel groups, with 83.3 ± 10.4% and 84.7 ± 12.2%, respectively, after 16 days.^20^ However, this result is similar to that of available wound treatment medicines.

Huang et al. achieved greater wound retraction using the TP3 peptide compared to the vancomycin group in wounds infected with multidrug-resistant bacteria on day 19 of the experiment (88.4 ± 7.89% and 76.6 ± 9.75%, respectively).^14^ Additionally, Li et al. achieved reductions of 99.1 ± 2.88% and 86.2 ± 10.50% in the NTSP and petroleum jelly, respectively, over 21 days.^22^

Liu et al., comparing TP2–5 peptide and Epidermal Growth Factor (*EGF*) obtain 84.75 ± 10.7% and 74.75 ± 12.5% wound retraction in 10 days, data that corroborate those obtained by Mukta et al. (2024) who observed 70% ± 8.7% and 44 ± 9.2% wound retraction in 7 days, comparing NTSP and bovine collagen, in same experiment wound retractions on the 10th day were 100% ± 0.0% and 89% ± 14.9% in the same groups.^4,31^

Ouyang et al. evaluated lyophilized collagen from NTSP and burn oil, achieving 83.50 ± 11.50% and 77.20 ± 12.53% wound retraction, respectively, within 14 days. The same author and his collaborators (2021) used the TP-2 peptide from tilapia skin and obtained 60.4 ± 4.29% compared to 54.8 ± 6.56% for burn oil in 14 days.^26,35^

Sastri et al. observed no difference in wound retraction in 7 days of the experiment, obtaining 46.23 ± 8.9% and 42.2 ± 9.5% when comparing NTSP and amniotic membrane, data that corroborate Tozetto et al., who obtained 39.75 ± 12.3% and 43.52 ± 9.6% comparing NTSP and bovine collagen on the seventh day of treatment, however same author observed 93.75 ± 10.69% and 71.5 ± 12 wound retraction on 14th day and 98.63 ± 14.34% and 88.2 ± 12.53% on the 21st day, both with a statistically relevant difference for the NTSP group concerning the control.^21,23^

Yang et al. did not observe any difference in wound retraction when comparing NTSP and bovine collagen treatments, with 79.39% ± 4.67% and 74.47% ± 6.50%; however, both were more expressive than the control (daily dressing with gauze) with 70.65% ± 4.21% in 14 days of experiment, also observed on the 21st day of treatment that both groups presented 100 ± 0.0% of wound retraction against 95.57% ± 0.24% of the control.^27^

Zhou et al. compared topical use of NTSP nanofibers with calcium and sodium alginate dressing (Kaltostat) obtained total scar retraction of 0.42 ± 0.11 and 0.62 ± 0.14 cm^2^ respectively, the same author Zhou and his collaborators (2016) compared NTSP sponge with (Kaltostat) obtaining total scar retration of 0.22 ± 0.06 and 0.38 ± 0.09 cm^2^, demonstrating greater scar retraction for NTSP group in both studies.^28,36^

##### 3.2.1.2 Inflammatory Cells and Blood Vessels

Cardoso et al. performed a histopathological analysis, which showed greater recruitment of inflammatory cells in the groups treated with NTSP (T1 and T2), with 10,440 ± 21,960 and 11,240 ± 2130 cells, respectively, compared to the control group, which had 9,275 ± 565 cells, on the ninth day. Additionally, the fraction of cells and blood vessels has increased in both T1 and T2 groups compared to the control group. Specifically, 7.53 ± 0.86% and 8.04 ± 1.04% of vessels were observed on the ninth day in groups T1 (NTSP) and T2, respectively, compared to 6.35 ± 0.77% in the control group.^24^

Similarly, Tozetto et al. demonstrated a more pronounced reduction in the inflammatory infiltrate and a significant modulation of angiogenesis during the proliferative phase on days 14 and 21, as evidenced by the formation of granular tissue and the presence of blood capillaries. More pronounced results of blood vessel count were observed in the two groups treated with NTSP, with 3.09 ± 1.05 and 2.83 ± 1.33 vessels/mm^2^, compared to 2.13 ± 1.12 vessels/mm^2^ in the control group on day 7. Additionally, harmful modulation in the recruitment of inflammatory cells was observed, with 3.75 ± 2.83 cells/mm^2^ in the NTSP group and 6.12 ± 4.12 cells/mm^2^ in the bovine collagen group on the seventh day, and 1.35 ± 1.23 cells/mm^2^ and 5.54 ± 3.12 cells/mm^2^ on the 14th day.^23^ This data corroborates the findings of Liu et al., who measured 2.6 ± 0.50% and 3.2 ± 0.80% of inflammatory infiltrate scores after 7 days of treatment with NTSP and bovine peptides.^4^

Still, Garrity et al. observed 0.015 and 0.005 vessels per mm^2^ when comparing NTSP with hydrocolloid gel on 16th day, data that corroborate those observed by Li et al., who compared the use of NTSP and bovine collagen, observing 24.6 ± 4.86% and 11.3 ± 3.83% of blood vessels on day seventh and 33.2 ± 3.27% and 22.5 ± 3.22% on day 14th.^20,22^

Additionally, Wang and collaborators obtained a higher count, with 45.0 points on the inflammatory cell score evaluation for both NTSP and bovine collagen groups and 25.0 points for the control treated with antiseptic after 14 days. In this study, both collagen groups promoted the growth of microvessels, which are essential for blood supply and aid in wound repair.^29^

##### 3.2.1.3 Fibroblasts and Collagen

Wang et al. observed a fibroblast count score of 35.0 points in the NTSP, compared to 18.0 points in the bovine collagen group, on the 14th day. Regarding the collagen measurement, the values were 9.05 ± 0.80% and 7.88 ± 0.33% for the treatment with NTSP sponge and topical antiseptic, respectively, while the control group showed smaller tissue density and collagen fiber thickness. The density of total type I collagen was significantly higher for the collagen groups than the self-healing group during all days of analysis.^29^

Tozetto et al. measured an average of 20.5 ± 7.5 and 13.7 ± 5.4 fibroblasts/mm^2^ in the NTSP and bovine collagen groups on the seventh day. The collagen fiber concentration in the NTSP group was 59.5 ± 4.6 fibers/mm^2^, whereas, for the bovine collagen group, it was 50.9 ± 5.1 fibers/mm^2^ on the 14th day of treatment.^23^

Li et al. also measured significantly different scores of 1.25 ± 0.25 for the group treated with NTSP and 0.82 ± 0.30 for the group treated with porcine collagen on the 21st day.^22^ Similarly, Ouyang et al. determined values of 5.06 ± 0.22% and 4.03 ± 0.39% collagen for the NTSP (TP-2) and Mebo commercial ointment groups on the 21st day.^26^ Sastri et al. observed a mean collagen density score of 8.25 in the NTSP group and 16.50 in the negative control, demonstrating regulation in the deposition process and resulting in a thinner scar with less fibrin deposition.^21^

##### 3.2.1.4 Immunohistochemical Analysis

Diverse authors evaluated the expression of immune markers involved in the phases of wound healing, including inflammation, proliferation, and remodeling. In this context, a greater expression of this gene was observed in treatments with NTSP. Elbialy et al. determined the expression of *TGF-β* at 71.95 ± 2.79 pg/mL and 40.975 ± 1.56 pg/mL, comparing NTSP gel with conventional gauze dressing on the 15th day, while Zou and collaborators determined the expression of *TGF-β* at 185.5 ± 29.7 and 139.4 ± 23.9 pg/mL, comparing treatment with NTSP nanofiber and conventional dressing on 28th day.^30,36^

Huang and collaborators observed in a study of wounds infected with *Staphylococcus aureus* multiresistant that topical treatment with vancomycin presented *IL-6* expression of 2.75 ± 0.23 pg/mL against 1.25 ± 0.39 pg/mL of treatment with NTSP TP-3 (an antimicrobial peptide isolated from Nile tilapia), demonstrating modulation of the inflammatory process in the TP-3 treatment.^14^ These data corroborate those determined by Ouyang and collaborators (2018), which showed a present modulation of the inflammatory response via *IL-6* expression in the NTSP TP-2 group, with 106.2 ± 11.76 pg/mL, compared to 126.2 ± 9.12 pg/mL for the Mebo group on the 21st day.^26^ Additionally, Garrity et al. (2020) measured *IL-6* expression at 0.77 ± 0.09 and 2.06 ± 0.22 in the treatment group compared to the NTSP and hydrocolloid gel in histological sections evaluated 16 days after treatment.^20^ The observed data demonstrate that treatments using NTSP block the *IL-6* expression pathway more effectively than conventional treatments, highlighting the promising effects of NTSP in controlling chronic conditions that are difficult to treat.

Huang et al. evaluated the expression of *TNF-α* in wounds of mice infected with *S*. *aureus* multiresistant and obtained 1.22 ± 0.35 mg/mL of expression for the group treated with NTSP TP-3 and 2.04 ± 0.78 mg/mL for the control with vancomycin on the third day.^14^ Ouyang and collaborators also evaluated the NTSP TP-2 compared to Mebo ointment and observed the expression of *TNF-α* at 46.2 ± 5.32 and 40.2 ± 7.22 mg/mL, respectively, on the 21st day.^26^ These results suggest a modulation in the inflammatory response mediated by the regulation of gene expression for *TNF-α* production in the groups treated with NTSP, compared to treatments available for complex wounds.

Elbialy et al. observed mean values and standard deviations for *VEGF* gene expression of 63.425 ± 4.4 pg/mg in the group treated with NTSP gel, compared to 35.825 ± 5.2 pg/mg in the group treated only with conventional gauze dressing, after 15 days.^30^ Garrity and collaborators determined *VEGF* expression at 1.97 ± 0.4 pg/mg and 0.95 ± 0.32 pg/mg for the intervention groups with NTSP and the control group with hydrocolloid gel on the 16th day.^20^ Evaluating the efficacy of lyophilized NTSP compared with commercial ointment for skin burns, Ouyang et al. (2018) determined the expression of *VEGF* at 56.2 ± 7.91 pg/mg and 47.3 ± 9.12 pg/mg, respectively, for these treatments with 14 days of the experiment.^26^ The same author (Ouyang) and collaborators (2021) measured *VEGF* expression at 212 ± 25 and 149 ± 21 pg/mg, comparing NTSP TP-1 with the commercial ointment Mebo after 21 days of topical treatment.^35^ These results suggest that treatments with NTSP more intensely stimulate the expression of the gene responsible for *VEGF* production.

Cardoso et al. observed the expression of the *CD31* gene, comparing topical treatment with the T19 NTSP (50 μL) to conventional treatment, which involved only cleaning the lesion and applying a dressing. They obtained 7060 ± 456 pixels mm^2^ and 5997 ± 227 pixels mm^2^, respectively, on the ninth day.^24^ Additionally, Liu et al. compared the treatment with NTSP TP-2(5) and *EGF* and obtained *CD31* expression levels of 3.12 ± 0.76 μg/mL and 2.42 ± 1.12 μg/mL, respectively, on the fourth day.^4^ Therefore, these results corroborate the hypothesis that NTSP activates the CD31 gene expression pathway in epithelial cells, significantly stimulating angiogenesis in groups treated with NTSP.

NTSP increases the expression of *FGF*, which is related to its ability to accelerate the healing process since Garrity et al. determined the expression of *FGF* on the 21st day of treatment at 1.05 ± 0.21 and 0.85 ± 0.12 μg/mL for the NTSP and hydrocolloid gel groups.^20^ Still, Ouyang and collaborators measured 164.5 ± 8.43 μg/mL and 129.2 ± 9.77 μg/mL for the NTSP groups compared to the commercial ointment after 14 days of topical application.^26^ Additionally, Wang et al. compared the treatment using a NTSP with a bovine collagen sponge, achieving 35.0 ± 8.0 and 18.0 ± 7.0 μg/mL of *FGF* expression on the 14th day.^29^

#### 3.2.2 *In Vitro* Studies

Of the total number of articles selected, four (*n* = 4) evaluated the antibacterial activity of NTSP, seven (*n* = 7) evaluated the cytotoxic activity in different cell lines, four (*n* = 4) evaluated cell migration through the scratch assay, and four (*n* = 4) evaluated delivery systems carrying NTSP.

##### 3.2.2.1 Antibacterial Activity

The antibacterial activity of TP3 was determined using the Minimum Inhibitory Concentration (MIC) method, which yielded a value greater than 3.9 μg/mL against multidrug-resistant *Staphylococcus aureus* (MRSA).^14^ Furthermore, TP3 at concentrations greater than 3.9 μg/mL effectively eliminated MRSA in 10 mM sodium phosphate buffer (pH 7.2). This study revealed that TP3, like other antimicrobial peptides, is unlikely to induce resistance and can be used as an adjunct to antibiotics, particularly as a prophylactic measure for situations with a high risk of infection.

Peptides obtained from the crude hydrolyzed extract of Nile tilapia skin also exhibited antibacterial efficacy through a bacterial growth inhibition assay. Various concentrations of the extract were tested (0.468–15 mg/mL). At a low concentration of 3.75 mg/mL, it inhibited *Escherichia coli* and *S*. *aureus* by more than 95.04 ± 1.08% and 91.90 ± 2.81%, respectively. These results suggest that this product may directly impact wound healing, particularly when the skin barrier is exposed to potentially pathogenic microorganisms.^23^

In another study, the antibacterial activity of NTSP, assessed by the zone of inhibition method, showed a minimal antibacterial effect against *E*. *coli* and *S*. *aureus*, with average inhibition zone diameters of 0.08 mm and 0.5 mm, respectively.^26^ In contrast, when these NTSPs were combined with chitosan to produce a hydrogel, they exhibited significant antibacterial activities, with average inhibition zones of 2.5 mm for *E*. *coli* and 4.0 mm for *S*. *aureus*. It suggests that conjugating NTSP to chitosan can preserve its bioactivity and enhance its antibacterial effect.^26^

Additionally, Ouyang et al. produced chitosan hydrogels incorporating NTSP, nanohydroxyapatite, and cross-linked with tannin. The antibacterial activity was determined using a colony counting approach, demonstrating excellent performance against *S*. *aureus* and E. *coli*. The variable in the hydrogel formulations was the concentration of nanohydroxyapatite, which showed no significant difference in the antibacterial test results, suggesting that the main activity originates from the NTSP and chitosan.^35^

##### 3.2.2.2 Cytotoxicity and Biocompatibility

The cytotoxicity of various TP3 concentrations was tested in BHK-21 cell cultures using neutral red uptake, LDH, and MTT assays. The results demonstrated that TP3 concentrations up to 40 μg/mL did not affect cell viability.^14^

The relative proliferation rate was calculated to evaluate the cytotoxicity of NTSP alone and after its incorporation into a chitosan-based hydrogel. This was done by comparing the optical density of the samples with that of the blank control (cell culture medium without the sample). The results showed cell viabilities of 104.0% for chitosan alone, 112.5% for NTSP, and 125.5% for hydrogels, indicating that all samples promoted the growth of L929 cells at a level greater than that of the control group.^26^

L929 mouse fibroblasts were cultured in contact with NTSP sponge at different concentrations, and the viability, growth, adhesion, and migration of the cells were subsequently observed by cell staining and scanning electron microscopy. The results showed a relative proliferation rate of more than 90%, with no cytotoxicity observed. In addition, many cells adhered to and migrated on the surface and within the NTSP sponge, with cell division and growth, indicating that the material exhibits excellent biocompatibility.^29^ Similarly, the viability and proliferative capacity of fibroblast cells cultured in lyophilized NTSP solution were tested. The viability rates were 100%, 75%, 50%, and 25%, and the relative proliferation rates were 24.34%, 97.33%, 87.68%, and 84.10%, respectively, demonstrating no cytotoxicity.^27^

The cytotoxicity of NTSP acellular dermal matrix (TS-ADM) was evaluated and compared with that of the positive control (0.05% (w/v) phenol solution), commercial porcine acellular dermal matrix (DC-ADM), and the negative control (medicinal polyethylene extract solution) groups. L929 cells grew very well in the NTSP group, and there was no significant difference in metabolic activity, as measured by the MTT assay, between the TS-ADM (105.3 ± 11.1%) and DC-ADM (103.6 ± 16.4%) groups. Both were higher than the negative control.^22^

Zhou et al. evaluated the viability of HaCaT keratinocytes after treatment with various concentrations of NTSP. No cytotoxic effects were observed at concentrations ranging from 7.8125 to 500 μg/mL.^28^ HaCaTs cells were also seeded on NTSP nanofibers, and after 24 h, the cells were firmly attached, evenly distributed, and exhibited excellent morphology. After 5 days, the cell proliferation rate reached 114%, indicating that NTSP nanofibers promoted cell adhesion and proliferation without cytotoxic effects. It is likely due to their nanostructure, high specific surface area, and hydrophilic nature, which promote cell growth.^28^

RAW 264 cells were treated with peptides of different hydrolysis times (0, 2, 4, 6, and 19 h). Cell viability improved as the hydrolysis time increased, with no cytotoxicity observed after exposing macrophage cells to the hydrolysate for 19 h.^24^ The potential use of nanohydroxyapatite/chitosan/NTSP skin peptides hydrogels was evaluated as a burn wound dressing, and cytocompatibility was determined by assessing HUVECs cell proliferation using the CCK-8 assay. The hydrogel containing 1% hydroxyapatite and 3% NTSP exhibited significantly higher cell viability compared to the blank control, indicating no cytotoxicity.^35^

##### 3.2.2.3 Scratch Assay

HaCaT cells were treated with NTSP (6.25 to 50.0 g/mL) compared with *rhEGF* (recombinant human epidermal growth factor). The low concentration (6.25 μg/mL) was not remarkable compared to the control group. However, at concentrations between 12.5 and 50.0 μg/mL, a significant wound closure effect was observed at 12, 18, and 24 h. Cell migration induced by 50.0 μg/mL was almost identical to that of the positive control, demonstrating that NTSP could induce HaCaT cell migration.^25^

The L929 cell line was used to evaluate the effect of free NTSP and its incorporation into a chitosan-based hydrogel on cell migration. The number of migrated cells in the NTSP treatment was significantly higher than in the control; however, it was lower than in the hydrogel treatment, supporting the synergistic role of peptides and chitosan in promoting wound closure.^26^

Murine fibroblast 3T3 cells were treated with different concentrations of crude hydrolyzed NTSP extract (12.5, 25, and 50 μg/mL) and compared with a blank control, the extract peptides influenced cell migration with 92.93 ± 1.49% gap reduction at 25 μg/mL after 24h, and 75.94 ± 3.89% closure with 18h of the experiment at 50 μg/mL.^23^

The effects of different NTSP hydrolysis times (0, 2, 4, 6, and 19 h) on the migration of RAW 264 macrophages were investigated by the scratch closure rate after 24 h of incubation. The nonhydrolyzed sample (corresponding to 0 h of hydrolysis) was the only one that did not show significant results compared to the control group, while all other samples enhanced cell proliferation and migration. Furthermore, the most significant migratory potential was observed in the sample hydrolyzed for 19 h compared to the other groups.^24^

##### 3.2.2.4 Delivery Systems to Carry NTSP

Thus, among the articles selected for this work, four (*n* = 4) of the included articles utilized the NTSP in association with innovative pharmaceutical formulations (Table 3). The gel formulations included in the meta-analysis (Table 3) were not discussed in this subsection because they represent conventional topical applications of NTSP without advanced delivery strategies. This section focuses specifically on innovative delivery systems to improve the stability, bioavailability, and controlled release of NTSP.

**Table 3:** Summary of Studies on Delivery Systems for Wound Healing

delivery system	carrier	evaluation	main results and conclusion	reference
Polymeric scaffolds - core–sheath	NTSP + poly(lactic acid) (PLA) and collagen + PLA-*g*-Vac (grafting vinyl acetate)	Cytotoxicity (Vero cell) Histopathological analysis	Average fiber diameter and pore area: 107 nm and 0.08 μm^2^ (collagen and PLA-*g*-Vac)	^31^
			Water contact angle measurements indicated higher wettability of the scaffolds, which is beneficial for promoting cell adhesion and growth.	
			Cytotoxicity analysis in Vero cell lines demonstrated biocompatibility, indicating that the scaffolds should not elicit detrimental responses in biological environments.	
			Histological evaluations in a rat model showed accelerated wound healing.	
Polymeric - Hydrogel	chitosan-tilapia peptides hydrogel	Antimicrobial activity	Significant antibacterial activity against *Escherichia coli* and *Staphylococcus aureus* increased L929 cell migration compared to the control group and individual components (chitosan or NTSP), demonstrating a synergy-promoting antibacterial effect, as well as enhanced cell proliferation and migration.	^26^
		Cytotoxicity L929	Significantly shorter wound healing period than the control and commercially available burn ointment groups.	
		Migration assay	Less inflammatory cell infiltration, accompanied by pronounced neovascularization and re-epithelialization, on day 7 is essential for effective wound healing.	
		Histological and Immunohistochemical analysis	Immunohistochemical analysis revealed that the expression of fibroblast growth factor 2 (*FGF2*) and vascular endothelial growth factor (*VEGF*) was significantly higher.	
Nanocomposite polymer-ceramic	Nanohydroxyapatite-chitosan- NTSP hydrogel	Hemolytic tests	Cross-linking improved their mechanical strength and enhanced their antibacterial properties against *Escherichia coli* and *Staphylococcus aureus* while maintaining good cytocompatibility and a reduced hemolysis rate in the NTSP group.	^35^
		Antimicrobial activity	The area of burn wounds treated with the hydrogels decreased faster than that in the control groups, with a higher healing rate at 7 days and mild inflammation and extensive angiogenesis at 14 days.	
		Cytotoxicity HUVECs	Hydrogels led to increased protein synthesis and hydroxyproline, enhanced collagen production, increased expression of wound-healing-associated factors such as *STAT3* and *VEGF*, and decreased inflammation-associated *TNF-α* and *IL-6*.	
		Histological and Immunohistochemical analysis		
Polymeric-nanofiber membrane	NTSP nanofibers	Cell proliferation and qRT-PCR (HaCaTs) Histopathological analysis	Electrospun tilapia collagen nanofibers exhibit biomimetic characteristics, including good mechanical properties, thermal stability, and biocompatibility.	^28^
			Average fiber diameter and pore area: 310 nm and 2.75 μm^2^.	
			The nanofibers significantly increased the proliferation and differentiation of human keratinocytes (HaCaT cells).	
			Improved wound closure compared with untreated controls and commercial dressing (Kaltostat).	
			Increase re-epithelialization and granulation tissue formation on days 7 and 14.	
			qRT-PCR analysis indicated upregulation of differentiation-related genes (involucrin, filaggrin, and *TGase1*) in keratinocytes cultured on collagen nanofibers, further supporting their role in promoting skin cell maturation.	

Zhou et al. employed the electrospinning technique to create NTSP nanofiber membranes cross-linked with glutaraldehyde, thereby improving thermal stability by increasing the collagen denaturation temperature and enabling their application in humans. Compared with commercial formulations, they demonstrated effectiveness *in vitro* in cultures of murine fibroblast L929 cells and human keratinocytes HaCaT cells, respectively.^28^

Researchers also used other materials for advanced dressings for skin wounds. Mukta et al. prepared core–shell scaffolds with poly­(lactic acid) by electrospinning that resembled the extracellular matrix, with nanometric dimensions, increasing the contact surface with the wound and accelerating the healing process through exudates absorption, exchanges of gas and fluid, as well as bacterial protection of PLA and proved that the scaffolds presented a high percentage of relative wound reduction concerning time (macroscopically) through the *in vivo* test.^31^

Another combination that also seemed advantageous in dressings was chitosan with NTSP. Using a simple mixing technique, Ouyang et al. produced a hydrogel to deliver NTSP, demonstrating synergistic activity with increased antibacterial effects against common pathogens and enhanced fibroblast proliferation *in vitro*.^26^ In 2021, further improving the NTSP delivery system in wound healing, the same research group developed a nanocomposite hydrogel using nanohydroxyapatite, chitosan, and NTSP. Ouyang et al. obtained a highly porous structure with interconnected pores, similar to the extracellular matrix, which is conducive to moisture retention and cell adhesion. They also made progress with the cross-linking of the hydrogel, improving its mechanical strength and antibacterial properties while maintaining good cytocompatibility and a reduced hemolysis rate. Additionally, they achieved superior results compared to commercial treatments in terms of scar retraction and healing time.^35^

### 3.3 Quantitative Results and Meta-Analysis

Table 4 summarizes the meta-analysis results for the analyzed variables in general and distinct groups, such as animal species, period, application, and comparator.

**Table 4:** Summary of Meta-Analysis Results^a^

variables	group			mean difference	CI 95% LCL UCL		*I* ^2^ e *p*-value of Heterogeneity		N comparisons	N studies
Scar retraction area (mm^2^)	General			0.21	–0.08	0.50		*I* ^2^ = 98.6% *p* < 0.001	28	5
	Species		Rats	0.24	–0.08	0.55		*I* ^2^ = 98.6% *p* < 0.001	24	3
			Rabbits	–0.14	–0.89	0.61		*I* ^2^ = 0.0% *p* = 0.870	4	2
	Period		Up to 7 days	–0.26	–0.43	–0.08		*I* ^2^ = 81.0% *p* < 0.001	14	5
			8–14 days	0.87	0.21	1.53		*I* ^2^ = 100.0% *p* < 0.001	8	2
			15–21 days	0.17	–0.11	0.44		*I* ^2^ = 86.0% *p* < 0.001	6	1
	Application		In nature	–0.22	–1.91	1.48		*I* ^2^ = 90.0% *p* < 0.001	2	1
			Gel	0.44	0.09	0.79		*I* ^2^ = 99.0% *p* < 0.001	18	1
			Liquid	–0.14	–0.89	0.61		*I* ^2^ = 0.0% *p* = 0.870	4	2
			Sponge	–0.48	–0.71	–0.24		*I* ^2^ = 95.0% *p* < 0.001	4	1
	Comparator		With the active ingredient	0.27	–0.29	0.82		*I* ^2^ = 98.6% *p* < 0.001	15	5
			Without the active ingredient	0.15	–0.11	0.40		*I* ^2^ = 96.0% *p* < 0.001	13	4
Wound retraction area (%)	General			10.52	6.99	14.05		*I* ^2^ = 88.3% *p* < 0.001	28	4
	Species	Mice		18.82	11.71	25.94		*I* ^2^ = 0.0% *p* = 0.500	2	1
		Rats		6.54	3.78	9.29		*I* ^2^ = 53.0% *p* = 0.090	6	1
		Rabbits		10.91	6.27	15.56		*I* ^2^ = 91.0% *p* < 0.001	20	2
	Period	Up to 7 days		6.18	3.34	9.02		*I* ^2^ = 33.0% *p* = 0.170	8	3
		8–14 days		14.85	8.71	21.00		*I* ^2^ = 78.0% *p* < 0.001	8	3
		15–21 days		11.00	2.81	19.20		*I* ^2^ = 96.0% *p* < 0.001	8	3
	Application	Liquid		11.74	7.41	16.06		*I* ^2^ = 90.0% *p* < 0.001	22	3
		Powder		6.54	3.78	9.29		*I* ^2^ = 53.0% *p* = 0.090	6	1
	Comparator	With the active ingredient		7.95	2.99	12.92		*I* ^2^ = 92.0% *p* < 0.001	12	2
		Without the active ingredient		12.79	7.89	17.70		*I* ^2^ = 82.0% *p* < 0.001	16	3
Wound closure (%)	General			6.71	4.30	9.13		*I* ^2^ = 88.8% *p* < 0.001	34	2
	Species	Rats		5.59	0.42	10.75		*I* ^2^ = 81.0% *p* < 0.001	6	1
		Swine		3.44	1.40	5.48		*I* ^2^ = 0.0% *p* = 0.935	12	1
		Rabbits		8.57	4.72	12.42		*I* ^2^ = 94.1% *p* < 0.001	16	1
	Period	Up to 7 days		3.57	2.06	5.07		*I* ^2^ = 56.6% *p* < 0.001	12	2
		8–14 days		6.44	3.39	9.50		*I* ^2^ = 33.2% *p* = 0.160	8	2
		15–21 days		15.07	8.55	21.59		*I* ^2^ = 96.0% *p* < 0.001	8	2
		More than 21 days		3.20	1.03	5.37		*I* ^2^ = 0.0% *p* = 0.880	6	1
	Application	Decellularized skin		4.25	2.03	6.47		*I* ^2^ = 46.0% *p* = 0.020	18	1
		Powder		8.57	4.72	12.42		*I* ^2^ = 94.0% *p* < 0.001	16	1
	Comparator	With the active ingredient		5.75	2.53	8.97		*I* ^2^ = 87.0% *p* < 0.001	17	2
		Without the active ingredient		7.73	4.08	11.37		*I* ^2^ = 89.0% *p* < 0.001	17	2
Inflammatory cells	General			–1.69	–2.29	–1.09		*I* ^2^ = 76.6% *p* < 0.001	18	1
	Period	Up to 7 days		–1.36	–2.61	–0.12		*I* ^2^ = 74.0% *p* < 0.001	6	1
		8–14 days		–2.40	–3.70	–1.11		*I* ^2^ = 88.0% *p* < 0.001	6	1
		15–21 days		–1.22	–1.52	–0.92		*I* ^2^ = 0.0% *p* = 0.980	6	1
	Comparator	With the active ingredient		–2.78	–3.55	–2.01		*I* ^2^ = 59.0% *p* = 0.010	9	1
		Without the active ingredient		–1.03	–1.29	–0.77		*I* ^2^ = 6.0% *p* = 0.380	9	1
IL-6	General			–0.13	–0.27	0.00		*I* ^2^ = 90.3% *p* < 0.001	24	1
	Period	Day 1		–0.08	–0.36	0.20		*I* ^2^ = 96.0% *p* < 0.001	8	1
		Day 3		–0.24	–0.45	–0.04		*I* ^2^ = 75.0% *p* < 0.001	8	1
		Day 5		–0.09	–0.32	0.14		*I* ^2^ = 76.0% *p* < 0.001	8	1
Inflammation score	General			–0.56	–0.96	–0.16		*I* ^2^ = 93.9% *p* < 0.001	8	1
	Species	Rats		–0.55	–1.06	–0.03		*I* ^2^ = 95.0% *p* < 0.001	6	1
		Swine		–0.64	–1.05	–0.22		*I* ^2^ = 0.0% *p* = 0.560	2	1
	Period	Day 7		–0.82	–1.21	–0.43		*I* ^2^ = 48.0% *p* = 0.170	2	1
		Day 14		–0.28	–0.93	0.37		*I* ^2^ = 94.0% *p* < 0.001	4	1
		Day 21		–0.94	–1.29	–0.59		*I* ^2^ = 0.0% *p* = 0.420	2	1
	Comparator	With the active ingredient		–0.27	–0.93	0.38		*I* ^2^ = 93.0% *p* < 0.001	4	1
		Without the active ingredient		–0.87	–1.09	–0.64		*I* ^2^ = 0.0% *p* = 0.400	4	1
TGF-β 1	General			24.40	14.94	33.85		*I* ^2^ = 75.7% *p* = 0.016	4	3
	Species	Rats		30.98	28.76	33.19			2	2
		Swine		19.30	11.20	27.40		*I* ^2^ = 0.0% *p* = 0.370	2	1
	Period	Day 14		19.30	11.20	27.40		*I* ^2^ = 0.0% *p* = 0.370	2	1
		Day15		30.98	28.76	33.19			1	1
	Application	Gel		30.98	28.76	33.19			1	1
		Decellularized skin		19.30	11.20	27.40		*I* ^2^ = 0.0% *p* = 0.370	2	1
	Comparator	With the active ingredient		15.00	2.60	27.40		*I* ^2^ = 57.0% *p* = 0.130	1	1
		Without the active ingredient		28.42	20.79	36.05		-	3	3
Blood vessels (%)	General			8.09	3.51	12.67		*I* ^2^ = 98.3% *p* < 0.001	8	2
	Species	Mice		1.42	0.92	1.92		*I* ^2^ = 19.0% *p* = 0.270	2	1
		Rats		10.86	6.48	15.24		*I* ^2^ = 97% *p* < 0.001	6	1
	Period	Day 7		12.82	10.86	14.78		*I* ^2^ = 0.0% *p* = 0.650	2	1
		Day 9		1.42	0.92	1.92		*I* ^2^ = 19.0% *p* = 0.270	2	1
		Day 14		13.77	7.79	19.75		*I* ^2^ = 94.0% *p* < 0.001	2	1
		Day 21		3.41	2.22	4.61		*I* ^2^ = 0.0% *p* = 0.630	2	1
	Application	Liquid		1.42	0.92	1.92		*I* ^2^ = 19.0% *p* = 0.270	2	1
		Decellularized skin		10.86	6.48	15.25		*I* ^2^ = 97.0% *p* < 0.001	6	1
	Comparator	With the active ingredient		9.04	3.20	14.88		*I* ^2^ = 97.0% *p* < 0.001	3	1
		Without the active ingredient		7.26	0.05	14.47		*I* ^2^ = 99.0% *p* < 0.001	5	2

a IC 95%: 95% Confidence Interval; *I* ^2^: *I* ^2^ statistic; UCL: Upper confidence limits; LCL: Lower confidence limits; N: sample size.

Through meta-analysis, wound retraction was observed, with a mean difference of 10.52% (95% CI, 6.99–14.05) between the intervention and comparator groups, with statistically significant superiority for the intervention groups (Figure 2). For this variable, high heterogeneity was observed between studies and was statistically significant (*I* ^2^ = 88.13%, *p* < 0.001; Table 4). The mean differences of wound retraction for days 7, 14, and 21 were 6.18%, 14.85%, and 11.00%, respectively, with heterogeneity values of *I* ^2^ = 33.0%, *p* = 0.170, *I* ^2^ = 78.0%, *p* < 0.001, and *I* ^2^ = 96.0%, *p* < 0.001 (Table 4).

**Figure 2:**
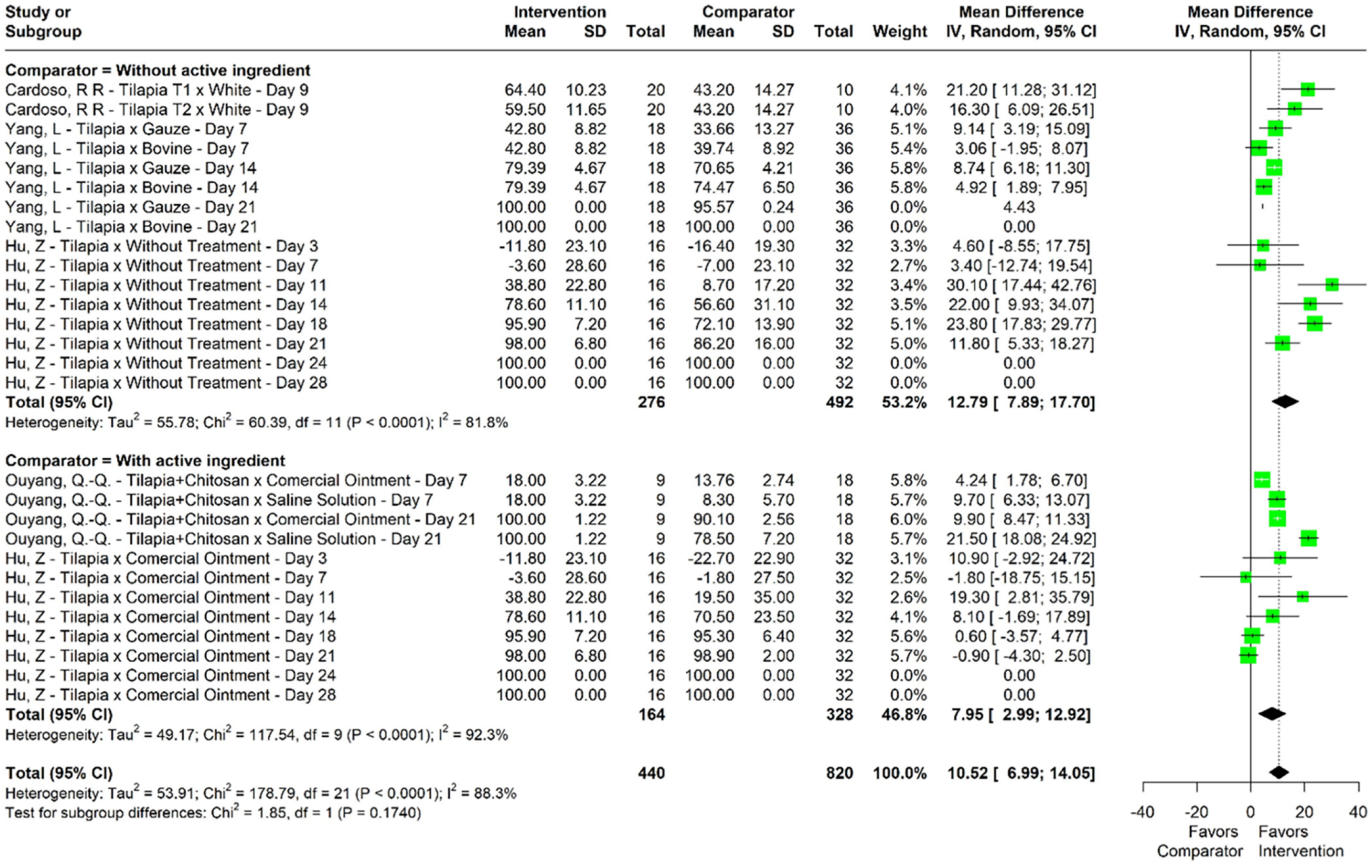
Forestplot of meta-analysis of mean difference for wound retraction (%).

The mean difference for scar retraction was 0.21 mm^2^ (95% CI – 0.08 to 0.50) in favor of the intervention group, indicating a trend toward greater retraction in studies using NTSP (Figure 3). However, heterogeneity across studies was also statistically significant (*I* ^2^ = 98.6%, *p* < 0.001; Table 4), which warrants cautious interpretation of these findings.

**Figure 3:**
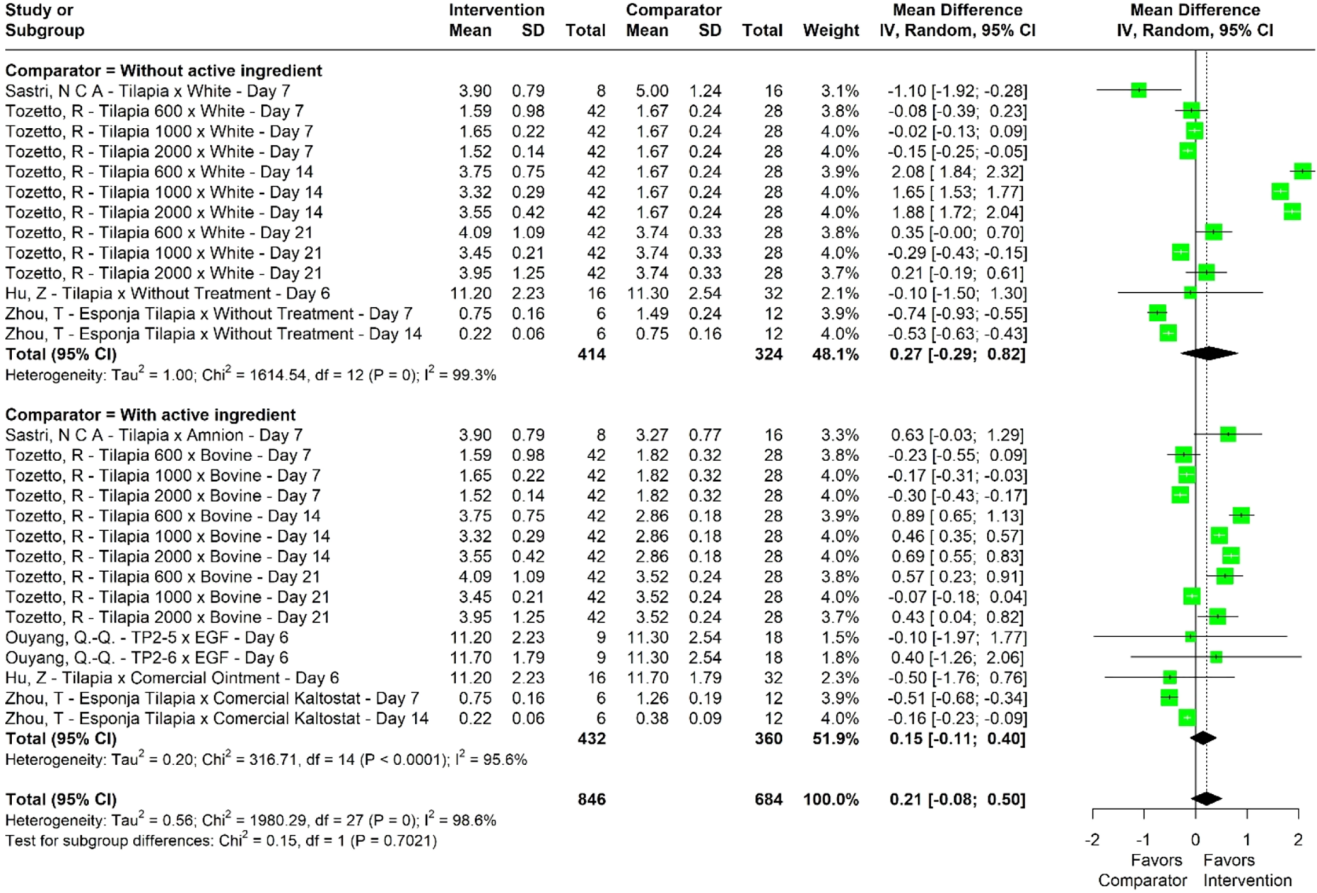
Forestplot of meta-analysis of mean difference for scar retraction (mm^2^).

The wound closure (%) was also determined, with an overall mean difference of 6.71% (*I* ^2^ = 88.8%, *p* < 0.001) between the intervention and comparator groups. For days 7, 14, and 21, an average difference of 3.57%, 6.44%, and 15.07% was determined, with heterogeneity values *I* ^2^ = 56.6%, *p* < 0.001; *I* ^2^ = 33.2%, *p* = 0.160; and *I* ^2^ = 96.0%, *p* < 0.001, respectively (Table 4).

Evaluation of inflammatory cell infiltration and score through meta-analysis indicated an overall mean difference of – 1.69 cells (95% CI, – 2.29 to – 1.09; *I* ^2^ = 76.6%, *p* < 0.001) and – 0.56 score points (95% CI, – 1.06 to – 0.03; *I* ^2^ = 93.9%, *p* < 0.001) between the intervention and comparator groups, respectively (Table 4). In the evaluated studies, lower mean counts of inflammatory cells and scores were observed in the intervention groups: – 1.36 cells for day 7 of the experiments, – 2.4 cells for the evaluations between days 8 and 14, and – 1.22 cells for day 21 (*p* = 0.217). For the inflammatory score, the mean difference was – 0.82 points on day 7, – 0.28 points on day 14, and – 0.94 points on day 21 (*p* = 0.212), as presented in Table 4.

In this context, for the expression of *IL-6*, an overall mean difference of – 0.13 pg/mL was obtained between the intervention and comparator groups in the studies with mice (*I* ^2^ = 90.3%, *p* < 0.001). The *IL-6* values for evaluations on day 1 were – 0.08, – 0.24 for day 3, and – 0.09 for day 5 (*p* = 0.515), as shown in Table 4.

Through meta-analysis, an overall mean difference of 8.09 vessels (95% CI 3.53 to 12.67; *I* ^2^ = 98.3%, *p* < 0.001) was observed between the intervention and comparator groups, with 1.42 and 10.86 vessels for mouse and rat studies, respectively, in favor of intervention treatments (*p* < 0.001). Meta-analysis also determined a mean difference of 12.82 vessels on the seventh day, 1.42 vessels on the ninth day, 13.77 vessels on day 14, and 3.41 vessels for evaluations on day 21, favoring the intervention (*p* < 0.001).

Studies included in this article that evaluated the expression of *TGF-β* using treatments with NTSP indicated an overall mean difference in *TGF-β* expression of 24.40 pg/mL (*I* ^2^ = 75.7%, *p* = 0.016). The average was 30.98 pg/mL in rats and 19.3 pg/mL in pigs, favoring the intervention groups (*p* = 0.006), as shown in Table 4. These results indicate that NTSP stimulates and activates the *TGF* growth factor expression pathways.

## 4 Discussion

This systematic review and meta-analysis of 16 preclinical studies demonstrate that NTSP improves wound closure, modulates inflammatory activity, and enhances neovascularization. However, substantial heterogeneity stemming from differences in extraction methods, dosing strategies, delivery systems, animal models, and assessment time points limits direct comparability and underscores the need for standardized experimental protocols and a thorough study of these bioactive compounds.

Diverse methods of peptide extraction are related, resulting in the isolation of different types of peptides. It also emphasizes the analysis of vital parameters for a comprehensive comparison and evaluation of the peptides extracted from Nile tilapia in wound management. Additionally, evaluating them in other clinically challenging wounds, such as burns and diabetic ulcers, using various animal models and clinical trials, is expected to expand knowledge and enhance the effectiveness of the peptides.

The parameters related to wound closure, such as scar and wound retraction, were the most frequently evaluated in the analyzed studies. It enabled more accurate comparisons in meta-analysis, particularly for inflammatory and angiogenesis variables. These are the primary and usual parameters evaluated during the wound healing process. Despite their description in the literature, the variability of animal study protocols makes it challenging to conduct a thorough comparison and discussion.

Specifically, our systematic revision and meta-analysis concerning wound closure indicate that NTSP generally performed better or at least equivalently compared with conventional treatments, including in burn and antibiotic-resistant infection models, with only a few exceptions.^20,21,23^ These effects may be attributed to peptide sequences rich in proline and alanine, which stimulate collagen synthesis, promote fibroblast chemotaxis, and enhance myofibroblast activity, thereby accelerating tissue retraction.^37^ Importantly, wound retraction and scar retraction represent distinct biological stages: the former is driven by early actomyosin-mediated contraction, and the latter by myofibroblast-dependent remodeling during the maturation phase, respectively.^38–40^

NTSP also contributes to the modulation of inflammatory responses, which is essential for successful tissue repair.^41^ Dysregulated inflammation, characterized by elevated proteases and prolonged neutrophil activity, can impair healing.^42,43^ NTSP supports the resolution of inflammation by reducing microbial burden, decreasing pro-inflammatory mediators, and promoting macrophage recruitment and polarization toward the M2 phenotype, which facilitates fibroblast proliferation and angiogenesis.^44^

There was a greater presence of blood vessels in all experiments that used NTSP, with a statistically significant difference compared to the controls. It demonstrates a relevant induction of neovascularization for the treatment, confirmed through meta-analysis, although high heterogeneity is observed among the studies, mainly due to differences in timing. NTSP increases vascular density and upregulates angiogenic mediators, including *FGF*, *VEGF*, *TGF-β*, angiogenin, and angiopoietins.^30,45,46^ These factors play a crucial role in oxygen delivery and granulation tissue formation, and newly formed vessels constitute up to 60% of the repair tissue.^47^

Fibroblast recruitment and ECM formation were also strengthened by NTSP, as evidenced by increased chemotaxis, *α-SMA* expression, and deposition of collagen and fibronectin.^25,48–52^ The proliferative phase is driven by fibroblast influx and *TGF-β1* activity, followed by macrophage polarization and keratinocyte migration, which initiate epithelialization and tissue deposition.^53,54^ Although some variability in collagen outcomes was reported, such as reduced type I collagen in one study, a thinner and more uniform scar suggested improved ECM regulation and remodeling.^55^ While the individual studies examined multiple molecular pathways, our meta-analysis revealed limited direct correlation between the mechanistic insights and the pooled clinical and histological outcomes, mainly due to heterogeneity in methodologies and reporting standards.

Immunohistochemical findings consistently show increased expression of *TGF-β*, *VEGF*, *FGF*, and *CD31* in NTSP-treated wounds, which are mediators central to the control of inflammation, endothelial proliferation, and tissue regeneration.^56–58^ However, across the included studies, inconsistencies in how these mediators were quantified or temporally assessed make it challenging to establish strong statistical associations between molecular activity and clinical end points. *TGF-β* orchestrates macrophage activation and fibroblast-to-myofibroblast differentiation, regulating *IL-1*, *IL-6*, and growth factor signaling essential for ECM synthesis.^59,60^ Modulation of *IL-6* and *TNF-α* is critical for preventing prolonged inflammation and chronic wound pathology, and NTSP appears to attenuate this cytokine activity.^61,62^ *VEGF*, *FGF*, and *CD31*, key regulators of endothelial migration and angiogenesis, were also consistently elevated; however, methodological variability limited the correlation with functional vascular metrics.^50,63–65^

*In vitro* evidence reinforces NTSP’s therapeutic potential, demonstrating biocompatibility, low cytotoxicity,^66^ and the capacity to promote the migration of fibroblasts and keratinocytes.^12,67^ NTSP also exhibited antimicrobial activity against *E*. *coli*, *S*. *aureus*, and MRSA, with enhanced effects observed when incorporated into chitosan-based hydrogels.^26,35^

Advanced delivery systems, including hydrogels, nanofibers, nanocomposites, and polymer-ceramic hybrids, further enhance the stability, release characteristics, and biological performance of NTSP, thereby accelerating wound closure while improving physicochemical properties.^35,68–70^ Continued development of these systems, aligned with regulatory standards, will be essential for clinical translation.

Overall, NTSP demonstrates strong potential as a bioactive therapeutic for wound management. However, progress toward clinical application requires standardized peptide formulations, sterility, and stability metrics, preclinical dose-escalation and pharmacokinetic/pharmacodynamic studies, the design of early phase (Phase I/II) clinical trials to evaluate safety, optimal dosing, harmonized study designs, robust biomarker reporting, and preliminary efficacy in well-defined wound types, such as burns and diabetic ulcers.

## 5 Conclusions

This review highlights the diverse roles of NTSP as an adjuvant biomaterial in wound healing. Evidence from selected studies on surgically induced wounds, burns, and scalds indicates that NTSP promotes biological activity and enhances healing outcomes by modulating inflammation and stimulating key cellular and molecular pathways, compared to other proposed treatments. Treatment with NTSP was associated with shorter healing times, greater wound closure rates, and reduced wound areas. These effects, corroborated by meta-analyses, also suggest a regulatory action on the inflammatory process, including the modulation of neutrophil and lymphocyte chemotaxis, as reflected in lower inflammatory scores in NTSP-treated groups. Additionally, NTSP treatment increased angiogenesis during the proliferative phase (days 15–21) and downregulated *IL-6* expression, accelerating the transition from inflammation to proliferation.

High biocompatibility and low toxicity of NTSP were also observed in both *in vivo* and *in vitro* studies. Importantly, it demonstrated efficacy even in wounds infected with multidrug-resistant microorganisms, underscoring its potential for the development of innovative dressings and treatments for complex wounds. By acting through multiple cellular and molecular pathways, NTSP offers distinct advantages over conventional therapies. Therefore, further investment in research, including clinical trials and novel delivery systems, is warranted to fully explore and translate the therapeutic potential of this biomaterial in regenerative medicine.

Although complex wounds such as diabetic and burn injuries involve distinct pathophysiological challenges, impaired angiogenesis, persistent inflammation, and oxidative stress, fundamental stages of wound healing and their underlying inflammatory signaling pathways remain conserved. This suggests a promising role for NTSP in such conditions. However, current evidence is limited to preclinical studies. Additional investigations, particularly in diabetic models, are crucial for confirming safety and efficacy, strengthening translational validity, and guiding clinical applications. Collectively, tilapia-derived peptides emerge as a promising therapeutic alternative; however, their clinical potential must be validated through further studies in relevant models.

## Abbreviations

NTSP: Nile tilapia Skin Peptides
ECM: Extracellular Matrix
*TNF-α*: Tumor Necrosis Factor-α
*IL*: Interleukin
*FGF-β*: Fibroblast Growth Factor-β
*VEGF*: Vascular Endothelial Growth Factor
*rhEGF*: Recombinant Human Epidermal Growth Factor
PLA: Poly­(lactic acid)
API: Active Pharmaceutical Ingredient
*Cox*: Cyclooxygenase-1
MMP: Matrix Metalloproteinase
*CD*: Cluster of Differentiation
*α-SMA*: α-Smooth Muscle Actin
*PCNA*: Proliferating Cell Nuclear Antigen
MRSA: Multi-Resistant *Staphylococcus aureus*
